# Predictive Cell Culture Time Evolution Based on Electric Models

**DOI:** 10.3390/bios13060668

**Published:** 2023-06-20

**Authors:** Juan Alfonso Serrano, Pablo Pérez, Paula Daza, Gloria Huertas, Alberto Yúfera

**Affiliations:** 1 Instituto de Microelectrónica de Sevilla (IMSE-CSIC), Av. Americo Vespuccio 24, 41092 Sevilla, Spain; jserrano10@us.es (J.A.S.); pablopg@imse-cnm.csic.es (P.P.); gloria@imse-cnm.csic.es (G.H.); 2 Departamento de Tecnología Electrónica, ETSII, Universidad de Sevilla, Av. Reina Mercedes sn, 41012 Sevilla, Spain; 3 Departamento de Biología Celular, Facultad de Biología, Universidad de Sevilla, Av. Reina Mercedes sn, 41012 Sevilla, Spain; pdaza@us.es; 4 Departamento de Electrónica y Electromagnetismo, Facultad de Física, Universidad de Sevilla, Av. Reina Mercedes sn, 41012 Sevilla, Spain

**Keywords:** bioimpedance, cell culture, computer-aided design (CAD), electric model, fractional order (FO), microelectrode, oscillation-based test (OBT)

## Abstract

Obtaining cell concentration measurements from a culture assay by using bioimpedance is a very useful method that can be used to translate impedances to cell concentration values. The purpose of this study was to find a method to obtain the cell concentration values of a given cell culture assay in real time by using an oscillator as the measurement circuit. From a basic cell–electrode model, enhanced models of a cell culture immersed in a saline solution (culture medium) were derived. These models were used as part of a fitting routine to estimate the cell concentration in a cell culture in real time by using the oscillation frequency and amplitude delivered by the measurement circuits proposed by previous authors. Using real experimental data (the frequency and amplitude of oscillations) that were obtained by connecting the cell culture to an oscillator as the load, the fitting routine was simulated, and real-time data of the cell concentration were obtained. These results were compared to concentration data that were obtained by using traditional optical methods for counting. In addition, the error that we obtained was divided and analyzed in two parts: the first part of the experiment (when the few cells were adapting to the culture medium) and the second part of the experiment (when the cells exponentially grew until they completely covered the well). Low error values were obtained during the growth phase of the cell culture (the relevant phase); therefore, the results obtained were considered promising and show that the fitting routine is valid and that the cell concentration can be measured in real time by using an oscillator.

## 1. Introduction

In recent years, a plethora of scholars have conducted research on monitoring the growth of a cell culture (CC) assay to develop noninvasive, cheap, and robust techniques [1,2,3,4,5,6,7,8,9]. Biomedical setups have included toxicology assays [10,11], cancer characterization experiments [12,13,14], biochemical experiments [15], immune assays [16], stem cell differentiation protocols [17], etc., and scholars have sought to use these setups to quantify the number of cells present to characterize a diversity of research objectives and techniques [18,19,20]. The modeling of a biological sample (BS) allows one to know its electrical behavior and several useful parameters. In many cases, these models are not useful to obtain the required result by themselves. For this reason, the main purposes of this experiment were to enhance the existing models and develop an automatic procedure to obtain the concentration of a CC in real time (RT).

The ECIS (electrical cell–substrate impedance sensing) technique is used to sense the electrical response generated on a BS ([21,22,23]), the CC, when it is excited with an alternating current or electrical voltage source at several frequencies due to its conductivity properties. The current–voltage relationship, or Ohm’s law, returns an impedance value with real and imaginary parts, the so-called bioimpedance (BI). Because the current applied to the BI must be very low, the ECIS technique requires precise and robust electronics [24]. Currents below 10 μA are employed to avoid any damage to the cells, and the applied voltage amplitudes are below 100 mV to correctly bias the electrodes in the linear region [5]. The CC is immersed in an ionic solution, the culture medium, and the CC cells settle on the substrate of the culture well, where the electrodes are placed. The ideal use of this technique is injecting a signal whose amplitude and frequency are set to their optimum values, i.e., the values that are most sensitive to a change in the BI. From this, the BI value is obtained, but to understand its value, a model of the electrical behavior of an electrode–cell system immersed in an ionic solution must be used. Some scholars have focused on modeling the BI by using solution electrical system equations [1,5] and performing finite elements simulations [25,26] of the whole system, which comprises the CC and electrodes.

**Figure 1:**
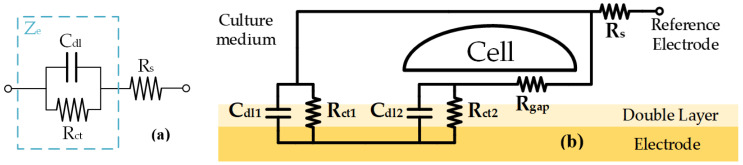
(**a**) Model of electrode immersed in an ionic solution. (**b**) Basic cell–electrode model.

The authors of reference [5] modeled the electrochemical behavior of an electrode immersed in an ionic solution. The electrical behavior of an electrode immersed in an ionic solution is modeled as the resistance of the electrode (*R_ct_*) in parallel with a capacitor (*C_dl_*), whose total impedance is *Z_e_* (Figure 1a). The spreading resistance (*R_s_*) is the opposition to the current flow in the saline solution that is in contact with the electrode. When the CC grows, it covers the electrode as a cell layer. The cell layer acts as an impedance whose resistive effects are added to the effect of the electrode. By using this model, the real electrical behavior of the cell–electrode (CE) system was reported in [27]. Figure 1b shows the layout of the electrical model components of a real CC. The electrical model contains the division of the *R_ct_* and *C_dl_* in two parallel branches, which models the electrode area (*A_e_*) covered by cells (*A_C_*) and not covered by cells (*A_nC_*). In this sense, the electrode is covered by cells (*A_C_*), which represents a measure of the CC time evolution, which is useful to determine the cell number and density.

Because real-time cell growth is the main parameter to be measured, the electrical model must include a parameter that acts as a cell-growth indicator. This parameter is the fill factor (*ff*), which is the percentage of the electrode area covered by cells in %1; i.e., if *A_c_* = *A_e_*, the *ff* value is 1 (confluence phase), but if *A_c_* = 0, the *ff* value is 0 (before seeding the cells at t = 0). Thus, *R_ct_*_1_, *C_dl_*_1_, *R_ct_*_2_, and *C_dl_*_2_ (the electrode model parameters covered and not covered by the cells, respectively) are defined by the following expressions:

(1) $$\begin{array}{c} R_{ct1}=\frac{R_{ct}}{1-ff}\;\;\;\;\;C_{dl1}=C_{dl}\cdot (1-ff)\\ R_{ct2}=\frac{R_{ct}}{ff}\;\;\;\;\;C_{dl2}=C_{dl}\cdot ff \end{array}$$

The transfer function (TF), which models the CE impedance, is obtained by solving the circuit in Figure 1b. Equation (2) shows the BI TF, *Z_CE_* (*s*), which is parameterized. It is described by using a parametrized second-order system, where ω_0*z*_ and *Q* are its natural frequency and quality factor, respectively. The constants *k*_0_, *k*_1_, and *k*_2_ are defined in Equation (2):

(2) $$\begin{array}{c} Z_{CE}(s)=\frac{k_{2}\cdot s^{2}+k_{1}\cdot \frac{\omega_{0z}}{Q}\cdot s+k_{0}\cdot \omega_{0z}^{2}}{s^{2}+\frac{\omega_{0z}}{Q}\cdot s+\omega_{0z}^{2}}\\ k_{2}=R_{s}\;k_{1}=R_{s}+\frac{R_{gap}\cdot R_{ct1}}{2\cdot R_{gap}+R_{ct1}+R_{ct2}}\;\;\;\;\;k_{0}=R_{s}+\frac{R_{ct1}\cdot (R_{gap}+R_{ct2})}{R_{gap}+R_{ct1}+R_{ct2}}\\ \omega_{0z}=\sqrt{\frac{R_{gap}+R_{ct1}+R_{ct2}}{R_{gap}\cdot {\left(C_{dl}\cdot R_{ct}\right)}^{2}}}\;\;\;\;\;Q=\omega_{0z}\cdot \frac{R_{gap}\cdot C_{dl}\cdot R_{ct}}{2\cdot R_{gap}+R_{ct1}+R_{ct2}} \end{array}$$

**Figure 2:**
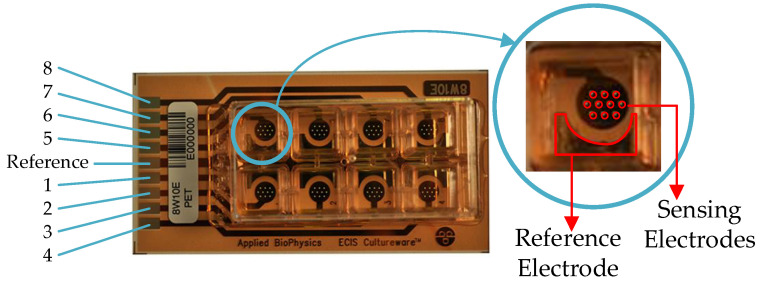
8W10E PET applied biophysics electrodes. Eight separated wells with ten circular biocompatible gold microelectrodes with 250 μm diameter and a large reference electrode with an area larger than each circular microelectrode by approximately 400 squared micrometers.

This basic model, the single-electrode well (SEW) model, considers the fact that under the cell, the electrodes are a big, unique electrode. We propose two more models (which are enhancements of the SEW model) of the electrode in Figure 2, which consider the real electrode composed by 10 microelectrodes and 1 large reference electrode: the real-electrode well (REW) and fractional-order well (FO REW) model. Enhanced electrodes with optimum sensitivities can be researched, as shown by the authors of [28].

This experiment is based on the experiments and measurements conducted by the authors of previous papers [27]. For these experiments, the measuring circuit was an oscillator. From the oscillation frequency and amplitude, which varies with *ff*, the cell concentration in real time (RT) can be obtained by using the Barkhausen stability criterion (BSC), which is the mathematical condition that the closed-loop feedback system must fulfill before obtaining sustained oscillations.

The objective of the presented experiment is to evaluate the impact of the proposed electric models on the cell cultures and electrodes in the assay and their time evolution in such a way that allows us to predict the cell number and density from the start time of the experiment until its confluence phase. We will then describe the electric models that we employed and the computer algorithms that we developed to create real-time predictions of the cell-culture status.

## 2. Material and Methods

The combination of model fitting, the use of an oscillation-based test (OBT) as a sensor, and the minimization of the cost function (CF) resulted in the *ff* values and the cell concentration in RT, i.e., during the real growth experiment of a CC assay, being predicted as the main targets. First, we designed a routine (based on previous work [29]) in which a real experiment is simulated and the *ff* value and the cell concentration is predicted at each moment. This routine will be tested by using the REW and FO REW electrical models on the data with three cell lines obtained from the experiment.

The first cell line was formed by Chinese hamster ovarian fibroblasts. This cell line was identified as AA8 (purchased from the American type culture collection), and this sample was immersed in McCoy’s medium supplemented with 10% (*v*/*v*) fetal calf serum, 2 mM L-glutamine, 50 μg/mL streptomycin, and 50 U/mL penicillin.

The second and third biological samples that were tested were two mouse neuroblastoma cell lines. The N2a cell line stably expressed a wild-type human amyloid precursor protein, N2a-APP. Both N2A and N2A_APP were provided by Dr. Javier Vitorica from IBiS (Instituto de Biomedicina de Sevilla) Sevilla (Spain). The cells were cultured in a medium consisting of 50% DMEM high glucose (Biowest, Nuaille, France) and 50% Opti-MEM (Gibco, Alcobendas, Spain) supplemented with 10% (*v*/*v*) fetal bovine serum (FBS) (Gibco), 2 mM L-glutamine, 50 μg/mL streptomycin, and 50 U/mL penicillin (Sigma-Aldrich, Madrid, Spain). N2a-APP was also supplemented with 0.4% geneticin (Gibco).

All the cell lines were maintained at 37 °C in a humidified atmosphere with 5% CO_2_, and they were routinely subcultured. Different initial numbers of cells (*N_ini_*) were seeded for our experiments: 2500, 5000, and 10,000 cells. The AA8 experiments started with a *N_ini_* of 2500 cells in wells 1 and 3, 5000 cells in wells 4 and 5, and 10,000 cells in wells 7 and 8. Moreover, the N2a and N2aAPP experiments began with a *N_ini_* of 2500 cells in wells 2 and 6, 5000 cells in wells 3 and 7, and 10,000 cells in wells 4 and 8. The plates contained eight separated wells with ten circular biocompatible gold microelectrodes with a 250 μm diameter each, which were designed for general-purpose cell culture applications. The electrodes employed in the cell culture assays (8W10E PET), whose electric model was employed for this experiment, were delivered by Applied Biophysics [30]. These electrodes are fabricated for ECIS equipment and are sold by this company. They are specifically designed for cell cultures and are employed in many assays that are referenced in the bibliography. The electrode size is defined by removing the top isolating mask. The selected size (250 μm diameter) is related to the common size of cells (1–100 μm diameter). The sensing area of the electrode is concentrated at the central part of the cultureware, and it obtains the maximum cell sensitivity (to avoid border effects). This layout has been enhanced by the same company, which delivers an IDE configuration (8W10E+ PET) that allows the sensing area of each well to be optimized. An optimized design can be obtained for each cell line, but this is not the purpose of this experiment. The electric models described here can be used for electrical simulation by considering the electrode area as the main parameter to define the final design. The ECIS technique relies on the attachment of the cells to the substrate, which is the electrodes for our experiment. So, a larger electrode area means a higher system sensitivity. This electrode area must be large enough to correctly sample the number of cells. From the datasheet of electrodes, 500–1000 cells can be sensed. This number must be the mean value considering the most common cell-size value, and the cell density must be homogeneous at every cultureware.

### 2.1. Bioimpedance Modeling

#### 2.1.1. Real-Electrode Well Model (11 Electrodes)

**Figure 3:**
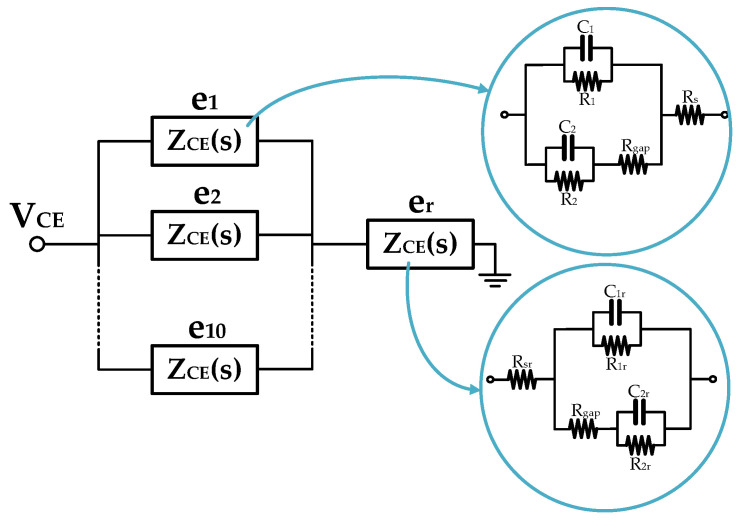
Real-electrode well (REW) model of the well. It has ten microelectrodes (*e*_1_ to *e*_10_) and one big reference electrode (*e_r_*).

The REW model contains the real electrode structure, ten microelectrodes, and one big reference electrode (Figure 2). Figure 3 shows a block diagram of the REW model between voltage *V_CE_* and the ground. Ten microelectrodes are present, *e*_1_ to *e*_10_, as well as the reference electrode *e_r_*. Each electrode is modeled after the electrode in Figure 1. The only difference is the value of the reference electrode parameters because this depends on the electrode area. To reduce the complexity of the model, the relationship between the microelectrode model parameters and the reference electrode model can be used (*R_gap_* is considered to have the same value for microelectrodes and reference electrodes). The relationship between *R_ct_* and *R_ctr_* (the reference electrode *R_ct_* parameter), between *C_dl_* and *C_dlr_* (the reference electrode *C_dl_* parameter), and between *R_s_* and *R_sr_* (the reference electrode *R_s_* parameter) can be derived from [5] as

(3) $$\begin{array}{c} \frac{R_{ctr}}{R_{ct}}=\frac{R_{ct}^{\prime }}{A_{re}}\frac{A_{e}}{R_{ct}^{\prime }}\to R_{ctr}=R_{ct}\frac{A_{e}}{A_{re}}[\Omega ]\\ \frac{C_{dlr}}{C_{dl}}=\frac{C_{dl}^{\prime }\cdot A_{er}}{C_{dl}^{\prime }\cdot A_{e}}\to C_{dlr}=C_{dl}\frac{A_{re}}{A_{e}}[F]\\ \frac{R_{sr}}{R_{s}}=\frac{\rho \cdot \sqrt{\pi }}{4\cdot \sqrt{A_{re}}}\frac{4\cdot \sqrt{A_{e}}}{\rho \cdot \sqrt{\pi }}\to R_{sr}=R_{s}\sqrt{\frac{A_{e}}{A_{re}}}[\Omega ] \end{array}$$

where *A_re_* is the reference electrode area and *ρ* is the electrolyte resistivity of the saline solution. Because the *A_e_*/*A_re_* factor is repeated, *k_e_* can be defined as *k_e_* = *A_e_*/*A_re_*. Therefore, the parameters of the electric model of the reference electrode are

(4) $$R_{ctr}=R_{ct}\cdot k_{e}\;\;\;\;{\;C}_{dlr}=\frac{C_{dl}}{k_{e}}\;\;\;\;R_{sr}=R_{s}\cdot \sqrt{k_{e}}$$

By using the relationship between the parameters of the microelectrodes and reference electrodes model, *Z_well_* (*s*) can be defined as the TF of the REW model. *Z_well_* (*s*) is composed of a gain, one pole, and one zero. The TF is very complex, but it can be simplified when *ff*→0 (the electrode does not have any cells) and *ff*→1 (the electrode is full of cells or is in the confluence phase), and one can obtain the pole, zero, and gain expressions for both cases:

(5) $$\begin{array}{c} p_{ff\to 0}=\frac{-1}{C_{dl}R_{ct}}\;\;\;z_{ff\to 0}=-\frac{R_{ct}\cdot (1+10\cdot k_{e})+R_{s}\cdot (1+10\cdot \sqrt{k_{e}})}{C_{dl}R_{ct}R_{s}\cdot (1+10\cdot \sqrt{k_{e}})}\\ p_{ff\to 1}=\frac{-1}{C_{dl}R_{ct}}\;\;\;z_{ff\to 1}=-\frac{R_{ct}\cdot (1+10\cdot k_{e})+R_{s}\cdot (1+10\cdot \sqrt{k_{e}})+11\cdot R_{gap}}{C_{dl}R_{ct}\cdot (R_{s}\cdot (1+10\cdot \sqrt{k_{e}})+11\cdot R_{gap})}\\ k_{well}^{ff\to 0}=R_{ct}\cdot \left(\frac{1}{10}+k_{e}\right)+R_{s}\cdot \left(\frac{1}{10}+\sqrt{k_{e}}\right)\\ k_{well}^{ff\to 0}=R_{ct}\cdot \left(\frac{1}{10}+k_{e}\right)+R_{s}\cdot \left(\frac{1}{10}+\sqrt{k_{e}}\right)+11\cdot \frac{R_{gap}}{10} \end{array}$$

By using the equations of poles and zeros, the *R_ct_* and *C_dl_* values when *ff*→0 and *ff*→1 are derived (because *p_ff_*_→0_ = *p_ff_*_→1_, only *p_ff_*_→1_ is used in the following expressions to reduce complexity and simplify the model fitting):

(6) $$\begin{array}{l} R_{ct}^{ff\to 0}=-\frac{R_{s}\cdot (1+10\cdot \sqrt{k_{e}})\cdot (p_{ff\to 0}-z_{ff\to 0})}{p_{ff\to 0}\cdot (1+10\cdot k_{e})}\\ C_{dl}^{ff\to 0}=-\frac{1+10\cdot k_{e}}{R_{s}\cdot (1+10\cdot \sqrt{k_{e}})\cdot (p_{ff\to 0}-z_{ff\to 0})}\\ R_{ct}^{ff\to 1}=-\frac{\left(R_{s}\cdot (1+10\cdot \sqrt{k_{e}})+11\cdot R_{gap})\cdot (p_{ff\to 0}-z_{ff\to 0}\right)}{p_{ff\to 0}\cdot (1+10\cdot k_{e})}\\ C_{dl}^{ff\to 1}=-\frac{1+10\cdot k_{e}}{\left(R_{s}\cdot (1+10\cdot \sqrt{k_{e}})+11\cdot R_{gap})\cdot (p_{ff\to 0}-z_{ff\to 0}\right)} \end{array}$$

To increase the fitting, the *R_s_* parameter is considered to change with *ff*, as described in [27]. Then, *R_s_* is split into two terms, *R_si_* and Δ*R_s_*, and $R_{s}\left(k\right)=R_{si}+ff^{n}\left(k\right)\cdot \Delta R_{s}$. The expression means that when *ff*→0, *R_s_* takes the value of *R_si_*, and the *ff* value on each moment *k* increases the *R_s_* value.

#### 2.1.2. Fractional Order Model

Fractional order (FO) models are based on the premise that the order of a differential operator can be a noninteger. The differential operator can be defined as the FO constant phase element (CPE), which is used to characterize electrodes for bioimpedance measurements of animal tissue. In combination with reference [31], the CPE can substitute the *C_dl_* term in the REW model to obtain the FO model. The capacitors of the CE model are replaced by the CPE and are described with FO operators:

(7) $$X_{C_{dl1}}=\frac{1}{s^{\alpha_{1}}C_{dl1}}\;\;\;\;\;X_{C_{dl2}}=\frac{1}{s^{\alpha_{2}}C_{dl2}}$$

where α_1_ and α_2_ are the FO orders of the reactance $X_{C_{dl1}}$ and $X_{C_{dl2}}$, respectively. Then, as in the previous section, the TF for the cases *ff*→0 and *ff*→1 can be obtained:

(8) $$\begin{array}{l} Z_{well}^{ff\to 0}(\lambda_{1})=k_{well}^{ff\to 0}\frac{p_{ff\to 0}}{z_{ff\to 0}}\frac{\lambda_{1}+z_{ff\to 0}}{\lambda_{1}+p_{ff\to 0}}\\ Z_{well}^{ff\to 1}(\lambda_{2})=k_{well}^{ff\to 1}\frac{p_{ff\to 1}}{z_{ff\to 1}}\frac{\lambda_{2}+z_{ff\to 1}}{\lambda_{2}+p_{ff\to 1}}\\ \mathrm{where}\;\lambda_{n}=s^{\alpha_{n}}\;0<\alpha_{n}<2 \end{array}$$

Note that when *ff*→0, α_2_ has no influence on the system behavior, and when *ff*→1, α_1_ has influence on the system behavior. Because changes in the α_1_ and α_2_ modify the magnitude slope and the frequency response phase, Δ*R_s_* ([27]) becomes redundant and can be removed from the FO model. Due to the high complexity of the model (the cross products of α_1_ and α_2_), the model is implemented in a different way than outlined in the previous section. The REW model is completely implemented to obtain its parameters, whereas the FO models are implemented in a transitional mode. Thus, considering that the pole is constant for any *ff* value, a transition from $z_{ff\to 0}$ to $z_{ff\to 1}$ and from $k_{well}^{ff\to 0}$ to $k_{well}^{ff\to 1}$ is implemented by using *ff* to change $Z_{well}^{ff\to 0}\left(\lambda_{1}\right)$ to $Z_{well}^{ff\to 1}\left(\lambda_{2}\right)$. Then, the implemented model is

(9) $$Z_{well}(\lambda_{1,}\lambda_{2})=k_{well}\frac{p}{z}\frac{\lambda +z}{\lambda +p}$$

where $p=p_{ff\to 0}=p_{ff\to 1}$ and

(10) $$\begin{array}{l} z=(1-ff)\cdot z_{ff\to 0}+ff\cdot z_{ff\to 1}\\ k_{well}=(1-ff)\cdot k_{well}^{ff\to 0}+ff\cdot k_{well}^{ff\to 1}\\ \lambda =(1-ff)\cdot \lambda_{1}+ff\cdot \lambda_{2} \end{array}$$

### 2.2. Cost Function

During the RT estimation, the previously described models utilize the oscillation frequency and amplitude values ($f_{CE}$ and $a_{CE}$) and return the *ff* and cell concentration values. In the next section, the process of using the model to predict *ff* in real time is explained, but a parameter (including *ff*) needs to be obtained from the BSC in some way. The BSC is the mathematical condition that the closed-loop feedback system must fulfill to obtain the sustained oscillations. An oscillator must meet some conditions to obtain self-sustained oscillation, which should include a linear part, *G*(*s* = *jω*) in the Laplace domain, and a nonlinear part, *N*(*a_osc_*, *f_osc_*), where *G* is the transfer function of the linear part of the circuit, *s* is the Laplace operator, *j* is an imaginary unit, *ω_osc_* is the oscillation frequency on *rad/s* whereby its value *ω_osc_* = 2*πf_osc_*, and *N* is the linearized model of the nonlinear part of the circuit (an electronic comparator for the present experiment). Note that *f_osc_* and *a_osc_* are not the same variables as $f_{CE}$ and $a_{CE}$, but *f_osc_* and *a_osc_* can be easily estimated by using some internal gains of the oscillator circuit. As the objective of this experiment is not to explain the oscillator circuit in detail, the calculation of the oscillating variables will not be explained in detail because its computation is trivial. Then, according to the BSC, the condition that the circuit must meet is

(11) $$f(a_{osc},f_{osc})\equiv 1+G(s=j\omega_{osc})\cdot N(a_{osc},f_{osc})=0$$

where *f*(*a_osc_*, *f_osc_*) is a complex function that depends on *f_osc_* and *a_osc_*. This function can be rewritten as

(12) $$f(a_{osc},f_{osc})=h_{1}(a_{osc},f_{osc})+j\cdot h_{2}(a_{osc},f_{osc})$$

where *h*_1_ and *h*_2_ are the real and imaginary parts of *f*(*a_osc_*, *f_osc_*). The main goal of using BSC is obtaining the oscillation parameters. Then, because the oscillation condition function must be equal to 0, in phasorial form, the condition is

(13) $$\begin{array}{c} f(a_{osc},f_{osc})\equiv h∠\varphi =0∠0{}^{\circ}\\ h=\sqrt{h_{1}^{2}+h_{2}^{2}}=0\\ \varphi =\tan^{-1}\left(\frac{h_{2}}{h_{1}}\right)=0{}^{\circ} \end{array}$$

where *h* and *φ* are the module and angle of *f*(*a_osc_*, *f_osc_*), respectively, and must be equal to 0 and 0°, respectively, to meet this condition. Equations of the real and imaginary part of the BSC now exist, which must be satisfied to obtain self-sustained oscillations. Using these equations, two parameters of the system could be obtained if all other parameters are known, but in this case, for the *ff* estimation process, obtaining these parameters is not possible. Therefore, the cost function is minimized to ensure that the BSC is satisfied so that the values of more than two of the model parameters can be obtained for each moment in combination with the fitting routine (which is outlined in the next section). The best way to meet the condition is to use the complex number *h*(*a_osc_*, *f_osc_*) module. Then, the CF can be defined as

(14) $$h(a_{osc},f_{osc})\equiv \sqrt{h_{1}{\left(a_{osc},f_{osc}\right)}^{2}+h_{2}{\left(a_{osc},f_{osc}\right)}^{2}}$$

The fact that *h*_1_(*a_osc_*, *f_osc_*) and *h*_2_(*a_osc_*, *f_osc_*) are squared assures that they cannot compensate each other.

### 2.3. Fitting Routine

**Figure 4:**
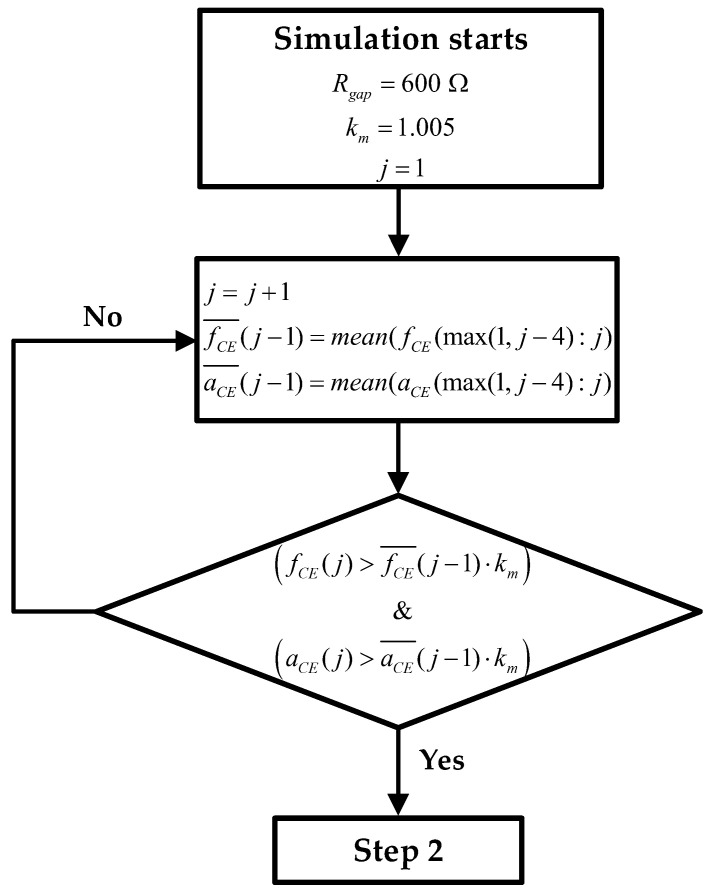
**Step 1**: block diagram of step 1 of RT simulation, whose target is to find the mean around the *f_CE_* and *a_CE_* minimums.

The key problem with knowing *ff* at each time during a real experiment is that the oscillation frequency and amplitude ($f_{CE}$ and $a_{CE}$) values are not available when the well is totally covered by cells (ff→1). That is, you can estimate the parameters of the models at the beginning of the experiment when any cells are in the well (ff→0), but certain parameters have no influence on the behavior of the system at this point. These parameters are *R_gap_*, $z_{ff\to 1}$, Δ*R_s_* (for the REW model), and α*_2_* (for the FO REW model). The designed routine is as follows:

1. **Estimate initial ***f_CE_*** and ***a_CE_*** values**: Figure 4 shows the block diagram of step 1, where the initial routine is presented in graphic form. During the first hours or days of the experiment, the mean of the last 5 $f_{CE}$ and $a_{CE}$ values is calculated ($\bar{f_{CE}}$ and $\bar{a_{CE}}$). As the sampling time (time between measurements) is 1 h, the average of the last 4 h is taken together with the values that were just obtained. After each measurement, after calculating the mean, a check is performed to verify whether the values obtained are greater than the mean of the new measurement plus a margin (*k_m_* = 1.005). If this condition is met, as shown in (15), the lowest $\bar{f_{CE}}$ and $\bar{a_{CE}}$ are stored as the minimum values. Figure 4 also defines the initial *R_gap_* value and the value of the constant *k_m_*. Note that the index *j* is the time index and goes from 1 to *j_max_*. When calculating $\bar{f_{CE}}$ and $\bar{a_{CE}}$, *j* is incremented from 1 until (15) is satisfied. *j_max_* is the maximum *j* value, and its value is defined by the number of measurements taken during the real experiment.

(15) $$\left(f_{CE}(j)>\bar{f_{CE}(j-1)}\cdot k_{m}\right)\&\left(a_{CE}(j)>\bar{a_{CE}(j-1)}\cdot k_{m}\right)$$

**Figure 5:**
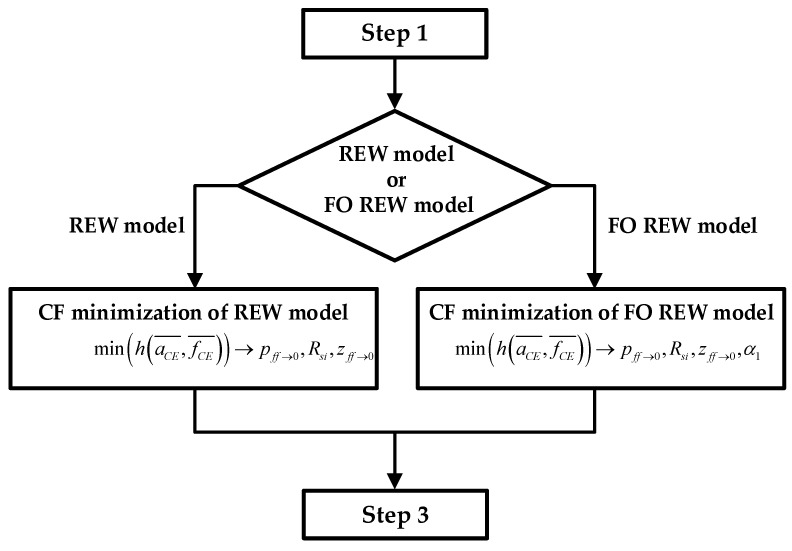
**Step 2:** block diagram of step 2 of RT simulation, whose target is to find the initial parameters (ff→0) by using the $\bar{f_{CE}}$ and $\bar{a_{CE}}$ values.

**Figure 6:**
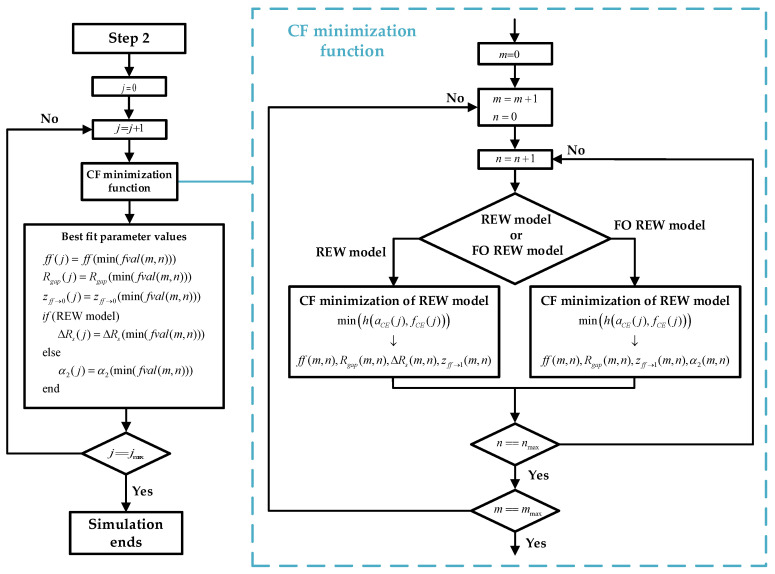
**Step 3:** block diagram of step 3 of RT simulation. It describes the simulation process from *j* = 1 to *j* = *j_max_* after obtaining the initial parameters.

2. **Computation of the initial parameters of the electrical models**: Using the minimum $\bar{f_{CE}}$ and $\bar{a_{CE}}$ estimated in the previous step, the initial parameters of the electrical models are fitted. The prediction is performed by using the CF minimization method. For the REW model, the parameters *p*_(*ff*→1)_ (whereby *p*_(*ff*→0)_ ≈ *p*_(*ff*→1)_), *R_si_*, and *z*_(*ff*→0)_ are calculated, and the *R_ct_*^(*ff*→0)^ and *C_dl_*^(*ff*→0)^ values can be derived from the parameters by using the two top equations of (6). For the FO REW model, the parameters *p*_(*ff*→1)_ (whereby *p*_(*ff*→0)_ ≈ *p*_(*ff*→1)_), *R_s_*, *z*_(*ff*→0)_, and *α_1_* are calculated, and the *R_ct_*^(*ff*→0)^ and *C_dl_*^(*ff*→0)^ values can be derived from the parameters by also using (6). The initial parameters that are calculated are the same for all *t*(*j*), and therefore, the values are not re-estimated during the simulation. The whole process of estimating the initial parameters is illustrated in the Figure 5 block diagram, which starts from the results of step 1 and ends at the beginning of the third and last step.
3. **Real-time *ff* estimation**: The last step and the goal of the routine is to predict the parameter *ff* in RT. Once the initial parameters of the models are obtained (after the previous step), *ff* is computed for all the previous measurements and the measurements that will be performed until the end of the experiment. Figure 6 describes the whole prediction process.

First, the time index *j* is initialized to start the *ff* estimation from *j =* 1 to *j* = *j_max_*. With the minimization, the model attempts to obtain the values of the parameters for each *j* measure: *ff*, *R_gap_*, *z*_(*ff*→1)_, and Δ*R_s_* (for the REW model) and *ff*, *R_gap_*, *z*_(*ff*→1)_, and *α*_2_ (for the FO REW model) by using $f_{CE}\left(j\right)$ and $a_{CE}\left(j\right)$. For this purpose, a loop is used to increment the index *j* from 1 to *j_max_*. Inside the loop, for each *j* value, the CF minimization function is used to obtain the candidate parameter values that will be used to obtain a lower *f_val_* value (minimum *h*(*a_osc_*, *f_osc_*) value). These candidate values are indexed by the indices *m* (from 1 to *m_max_*) and *n* (from 1 to *n_max_*); the *m* and *n* index are internal to the CF minimization function, so the parameters computed inside the function (*ff*, *R_gap_*, etc.) indexed with *m* and *n* are not the same as the parameters outside the function. When the parameters with the best *f_val_* are obtained, the parameters with the lowest *f_val_* are chosen and assigned as the values taken by the parameters for time *j*. Inside the CF minimization function, the first step is to define the bounds of these parameters, which are shown in (Section 2.2). The *ff* bounds change with each *j* estimation, but the bounds of the other parameters remain constant for all *j* estimations depending on the outcomes of the procedures outlined in the previous sections of this article.

(16) $$\begin{array}{c} ff^{bounds}(j)=\left[\begin{array}{cc} f(j-1)-0.2 & ff(j-1)+0.2 \end{array}\right]\\ R_{gap}^{bounds}=\left[\begin{array}{cc} 0.1 & \infty \end{array}\right]\;\;\;\;z_{ff\to 1}^{bounds}=\left[\begin{array}{cc} 10^{3} & 10^{5} \end{array}\right]\\ \Delta R_{s}^{bounds}=\left[\begin{array}{cc} -R_{s}^{ff\to 0} & 4\cdot R_{s}^{ff\to 0} \end{array}\right]\;\;\;\;\alpha_{2}^{bounds}=\left[\begin{array}{cc} 0.9 & \alpha_{1} \end{array}\right] \end{array}$$

The CF minimization is performed by using the values shown in (Section 2.3) as the initial values. As can be seen, $R_{gap}^{ini}$ and $z_{ff\to 1}^{ini}$ have four and two initial values, respectively. The index *m* moves along the vector $R_{gap}^{ini}$ (from 1 to *m_max_* = 4), and the index *n* moves along $z_{ff\to 1}^{ini}$ (from 1 to *n_max_* = 2). This is because for each *j* estimation, several CF minimizations are performed to obtain as many combinations of the initial values as possible, i.e., eight minimizations. The main purpose of this approach is to find the minimization that results in the lowest *f_val_* in a robust and computationally time efficient way. As a result, a matrix of values is obtained at each time *j* for each of the estimated parameters. This process is performed for each *j* value, after which, when finishing the function and as already explained, the values of the parameters for which *f_val_* is minimum are chosen (note that an *f_val_* exists for each value of the *m* × *n* matrix, and an *m* × *n* matrix exists for each *j* value).

(17) $$\begin{array}{c} ff^{ini}(j)=ff(j-1)\\ R_{gap}^{ini}=\left[\begin{array}{cccc} 10 & 100 & 10^{3} & 10^{4} \end{array}\right]\\ z_{ff\to 1}^{ini}=\left[\begin{array}{cc} 10^{3} & 10^{4} \end{array}\right]\\ \Delta R_{s}^{ini}=0\alpha_{2}^{ini}=1 \end{array}$$

The steps described above were applied for each *j* value of each well of each cell line, whereby we performed simulations that did not consider the future *f_CE_* and *a_CE_* values by using the REW and FO REW models. The following section shows the results of the simulation of the real CC assay experiments, which predicted the cell concentration in RT.

## 3. Results

The RT simulation method was designed to be implemented in a prototype model so that the *ff* and cell concentration values could be reported after each measurement. As the required sampling time was 1 h, the time taken to perform all the necessary mathematical operations was not a critical concern. Thus, the time taken to compute the initial parameters, *ff*, and other parameters for each measurement was not a critical problem.

The metric used to determine the accuracy of the method was the error in the cell concentration calculation. By using traditional optical counting methods, the cell concentration could be obtained (defined as *C_trad_*), whereby we obtained one concentration value per day (a time step of 24 h). *C_trad_* was compared with the cell concentration obtained in RT (*C_sim_*). *C_sim_* was obtained by using the following expression:

(18) $$C_{sim}(j)=\frac{\left(ff_{sim}(j)\frac{A_{well}}{A_{c}}\right)}{A_{well}}$$

where *ff_sim_* is the vector of the *ff* values obtained from the RT simulation, *C_sim_* is the cell concentration calculated by using *ff_sim_*, *A_c_* is the cell area, and *A_well_* is the well area. Comparing *C_trad_* with the ideal concentration (*C_i_*) is also desirable, which would have been performed if any errors occurred during the *ff* real-time estimation. The error in the *ff* estimation was measured with respect to the deviation from its ideal final value. For the maximum *ff* obtained during the simulation, the error is the difference between the maximum value obtained and the maximum value that the *ff* should reach, i.e., a value of 0.99. Thus, calculating the real *ff* curve that should have been obtained for each well is possible by using the following equation:

(19) $$\begin{array}{c} ff_{i}(j)=k_{ff}\cdot ff_{sim}(j)\\ k_{ff}=\frac{0.99}{\max\left(ff_{sim}\right)} \end{array}$$

where *ff_i_* is the vector of the theoretically real *ff* values and *k_ff_* is the factor applied to *ff_sim_* to obtain *ff_i_*. Then, *C_i_* is derived as follows:

(20) $$C_{i}(j)=\frac{\left(ff_{i}(j)\frac{A_{well}}{A_{c}}\right)}{A_{well}}$$

The metrics used to measure accuracy are the mean relative error on the cell concentration (*e_rm_*), in percent (%), defined as follows:

(21) $$\begin{array}{c} e_{rm.sim}=\sum_{j_{trad}}\frac{\left|C_{sim}(j_{trad})-C_{trad}(j_{trad})\right|}{C_{trad}(j_{trad})}\\ e_{rm.i}=\sum_{j_{trad}}\frac{\left|C_{i}(j_{trad})-C_{trad}(j_{trad})\right|}{C_{trad}(j_{trad})} \end{array}$$

where *j_trad_* is the index of the cell concentration obtained by using a traditional optical method.

**Figure 7:**
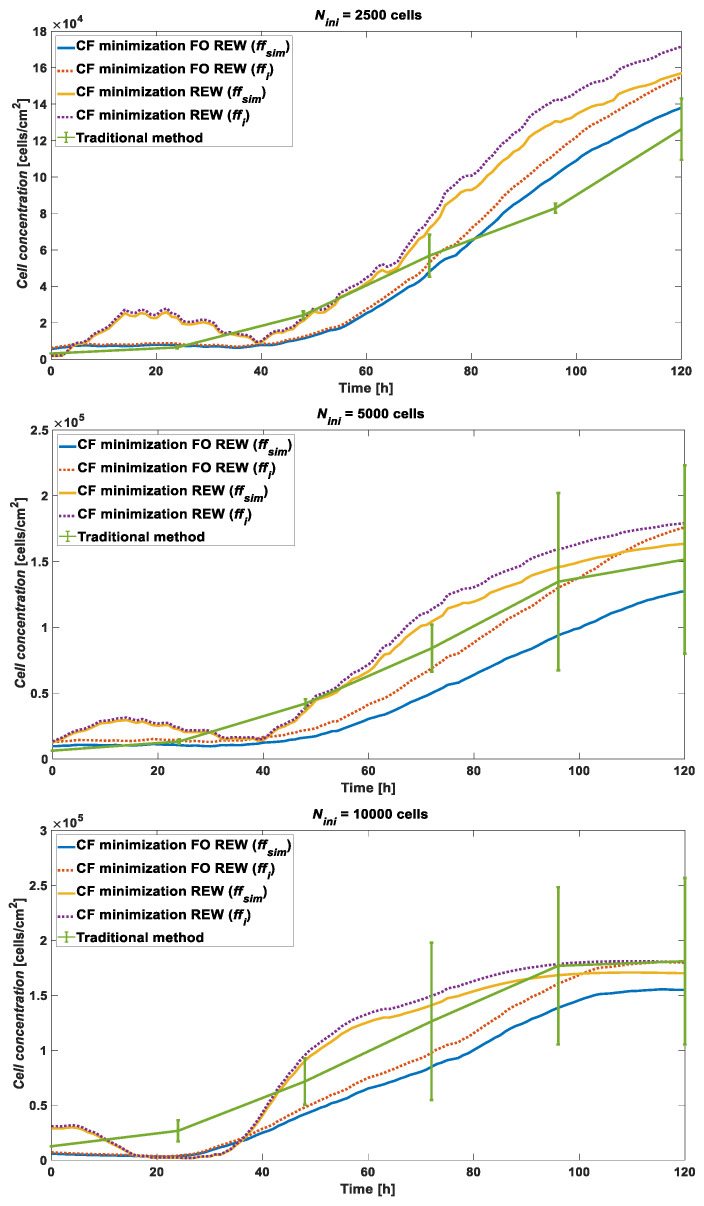
AA8 cell line cell concentration comparison between RT simulation using FO REW model (obtained from *ff_sim_* in blue and *ff_i_* in red), REW model (obtained from *ff_sim_* in yellow and *ff_i_* in purple), and traditional counting method (green).

Figure 7 shows the estimated cell concentration of the RT simulation for *N_ini_* with 2500, 5000, and 10,000 cells by using the experimental data obtained from the AA8 cell line. The blue and red lines represent the cell concentrations obtained by using the *ff_sim_* and *ff_i_* of the FO REW model, respectively. The yellow and purple lines illustrate the cell concentrations obtained by using the *ff_sim_* and *ff_i_* of the REW model, respectively. The last line, in green, represents the cell concentration obtained by using the traditional optical counting method. As can be seen, the FO REW model performed a little worse than the REW model on the final point (*t* = 120 h). In addition, the graph also shows that the higher the initial concentration, the more accurate the cell concentration estimation method.

The main results (and conclusions) are obtained from the mean relative error data. Thus, the RT simulation results of the three cell lines used during the present experiment (AA8, N2a, and N2aAPP) must be compared. The data were analyzed and compared and were divided into sections of the experiment. Two main sections (time windows) of interest existed: the initial section, where the cells adapted to the culture medium and adhered to the bottom of the well (*ff*→0), and the section from the beginning of the growth phase (the exponential phase) to the saturation phase of the well (*ff*→1).

Table 1 shows the *e_rm_* of the cell concentration for the three cell lines obtained throughout the whole experiment.

Table 2 shows the *e_rm_* of the cell concentration for the three cell lines. The error shown is the *e_rm_* of the cell concentration curves during the first hours and/or days of the experiment, i.e., from the time that the CC assay was seeded until the moment when it started to considerably grow (the beginning of the exponential phase). During the first simulation period, *e_rm_* was larger than the total *e_rm_* (Table 1). Specifically, the REW model returned much larger errors than the FO REW because it did not obtain strong results for the low *ff*.

The opposite occurred for the *e_rm_* during the second part of the simulation, which is detailed in Table 3. The *e_rm_* in the second half of the simulation was much lower than in the first half. The most notable difference was found for the simulations that used the REW model, because a large difference in error existed between the first and second frame, with the second one providing much stronger results (at the accuracy level of the FO REW model). Another point to note is that, in general, more accurate results are obtained at a higher *N_ini_*. Finally, the first simulation section is not as important in terms of predicting the *ff* and cell concentration. Therefore, these data are quite acceptable and provide a useful starting point to enhance the models and the simulation and parameter computation method.

A separate analysis of the two experiment zones provided interesting results. The cell concentration of a CC assay can be estimated in RT by using the CC assay during an OBT. The errors are still large, but with some enhancements to the algorithm, and of the OBT measurement prototypes, the error should be greatly reduced. Additionally, the *e_rm_* obtained in terms of cell concentration depends on the cell line because the lines with a lower *A_c_* (N2a and N2aAPP) reach much larger error values than the cell line with a higher *A_c_* (AA8).

## 4. Conclusions

A method used to minimize CFs was used to obtain the optimal values of the model parameters that meet the BSC for each *f_CE_* and *a_CE_* value. Due to the complexity of the used models (REW and FO REW), achieving sufficiently well-fitting results is not easy because a considerable number of parameters need to be calculated at each moment, and small variations in one of the parameters can cause the value of the *ff* parameter to differ from its real value. In addition, the *f_CE_* and *a_CE_* data for some cell lines were worse than the data for others due to small amplitudes in the voltage signals, as the prototype measurement was still in the experimental phase. Even with these difficulties, we successfully estimated the RT cell concentration present in a CC assay, although with a certain margin of error. One must be mindful of the variability in the cell concentration from well to well and the mismatching between electrodes. Every time the cells are seeded, the real number of cells that are being seeded can change according to the manual method followed. Thus, possible variabilities in the traditional cell culture may exist from well to well and from well to Petri plate. The factor to be considered is the mismatching of the electrode geometry during fabrication, which can generate deviations from the expected sensing area (π × radius^2^) and consequently change the actual electrode impedance. The deviations from the expected values were not small, and they were within the error bars in most cases. Considering the results of studies performed up to now, our results are promising and strong; our method is useful and should be enhanced by future studies.

We showed that cell concentrations can be obtained in real time during cell growth experiments by employing the methodology that we describe. The errors found, although considerable, can be reduced by enhancing the measurement circuit and the algorithm used to calculate the cell concentration.

## Figures and Tables

**Table 1:** Cell concentration mean relative error in %.

*N_ini_*	2500			5000			10,000		
Line	AA8	N2a	Na2APP	AA8	N2a	Na2APP	AA8	N2a	Na2APP
$e_{rm.sim}^{REW}[\%]$	59.37	372.52	396.43	37.56	154.33	219.13	46.40	83.80	25.51
$e_{rm.i}^{REW}[\%]$	68.47	430.92	443.78	46.70	173.84	271.15	49.79	91.49	22.31
$e_{rm.sim}^{FO\;\;REW}[\%]$	33.34	205.13	238.65	36.79	100.82	102.22	40.70	56.00	45.56
$e_{rm.i}^{FO\;\;REW}[\%]$	41.04	275.54	276.67	31.61	129.35	135.65	31.19	55.11	30.58

**Table 2:** Cell concentration mean relative error in % before to start the CC assay growth (*t* < 40 h).

*N_ini_*	2500			5000			10,000		
Line	AA8	N2a	Na2APP	AA8	N2a	Na2APP	AA8	N2a	Na2APP
$e_{rm.sim}^{REW}[\%]$	120.6	1082.9	946.0	88.8	390.4	515.9	111.1	200.2	31.4
$e_{rm.i}^{REW}[\%]$	128.9	1252.4	1056.6	101.1	472.5	616.7	118.6	230.6	25.6
$e_{rm.sim}^{FO\;\;REW}[\%]$	52.5	518.6	535.4	38.5	176.4	229.2	69.7	78.9	62.2
$e_{rm.i}^{FO\;\;REW}[\%]$	68.3	771.4	629.5	55.2	327.9	295.9	64.1	117.4	52.5

**Table 3:** Cell concentration mean relative error in % after starting the CC assay growth (*t* > 40 h).

Line	AA8	N2a	Na2APP	AA8	N2a	Na2APP	AA8	N2a	Na2APP
$e_{rm.sim}^{REW}[\%]$	28.7	17.3	30.0	11.9	36.3	21.3	14.0	25.6	21.6
$e_{rm.i}^{REW}[\%]$	38.3	20.2	35.2	19.5	24.5	40.8	15.4	21.9	20.1
$e_{rm.sim}^{FO\;\;REW}[\%]$	23.8	48.4	40.8	35.9	63.0	17.6	26.2	44.5	34.5
$e_{rm.i}^{FO\;\;REW}[\%]$	27.4	27.6	41.4	19.8	30.1	28.8	14.8	23.9	15.9

## Data Availability

All the experimental data were measured from cell cultures supported by this project. The data are available if required.
